# Dual Therapeutic Impact of AXL Inhibitor AB-329: Chemotherapy Sensitization and Immune Microenvironment Reprogramming in TNBC

**DOI:** 10.3390/ijms26188896

**Published:** 2025-09-12

**Authors:** Dileep Reddy Rampa, Jon A. Fuson, Huey Liu, Max Pan, Yujia Qin, Youping Deng, Naoto T. Ueno, Jangsoon Lee

**Affiliations:** 1 Preclinical Core, Cancer Biology Program, University of Hawaiʻi Cancer Center, Honolulu, HI 96813, USA; 2 Department of Breast Medical Oncology, The University of Texas MD Anderson Cancer Center, Houston, TX 77030, USAhuey.liu@mdanderson.org (H.L.); 3 Nuvation Bio Inc., New York, NY 10036, USA; max.pan@nuvationbio.com; 4 Department of Quantitative Health Sciences, John A. Burns School of Medicine, University of Hawaiʻi at Manoa, Honolulu, HI 96813, USAdengy@hawaii.edu (Y.D.); 5 Translational Clinical Research, University of Hawaiʻi Cancer Center, Honolulu, HI 96813, USA

**Keywords:** triple-negative breast cancer, AXL, AB-329, chemotherapy, tumor immuno-microenvironment, natural killer cells

## Abstract

AXL, a receptor tyrosine kinase, has emerged as a promising therapeutic target in triple-negative breast cancer (TNBC) due to its critical roles in tumor progression, metastasis, and immune evasion. In this study, we investigated the antitumor efficacy and immunomodulatory potential of AB-329, a selective AXL kinase inhibitor, in preclinical models of TNBC. Transcriptome analysis and single-cell RNA sequencing datasets revealed elevated AXL expression in mesenchymal TNBC subtypes and a negative association with immune cell infiltration. While AB-329 demonstrated moderate antiproliferative effects as a monotherapy, its combination with paclitaxel led to substantially enhanced antiproliferative and anti-metastatic effects compared to gemcitabine, DXd, and SN-38. In murine TNBC allograft models, the combination of AB-329 and paclitaxel significantly reduced tumor growth, and AB-329 increased activated natural killer (NK) cell infiltration in humanized mouse models. Analysis of human breast cancer tissue further confirmed that low AXL expression is associated with a higher presence of NK cells in the tumor. These findings suggest that AB-329 not only augments chemotherapy efficacy but also reshapes the tumor immune microenvironment, supporting its further development as a dual-action therapeutic strategy for AXL-positive TNBC.

## 1. Introduction

Triple-negative breast cancer (TNBC) is an aggressive subtype of breast cancer characterized by the lack of estrogen receptor (ER), progesterone receptor (PR), and human epidermal growth factor receptor 2 (HER2) expression. Representing approximately 15–20% of all breast cancer cases, TNBC disproportionately affects premenopausal women under the age of 40 [1]. Clinically, TNBC is associated with a poor prognosis, marked by a 40% mortality rate within five years of diagnosis and a high likelihood of early distant metastasis [2,3]. Even after surgical resection, recurrence rates remain significant (~25%), and mortality following recurrence can exceed 75% within three months [4,5]. These statistics underscore the urgent need for more effective and durable treatment strategies.

Advancements in molecular profiling have shown that TNBC is a highly diverse disease. Initial work by Lehmann et al. identified six molecular subtypes—basal-like 1 (BL1), basal-like 2 (BL2), mesenchymal (M), mesenchymal stem-like (MSL), immunomodulatory (IM), and luminal androgen receptor (LAR) [6,7]. Later research refined this into four main subtypes: basal-like immune-activated (BLIA), basal-like immunosuppressed (BLIS), mesenchymal-like (MES), and LAR [8,9]. Even with these classification advances, TNBC still has the fewest targeted therapy options among breast cancer subtypes. Many patients benefit only slightly from current treatments and often develop resistance, highlighting the need to find new molecular drivers of progression and resistance.

One such driver is AXL, a receptor tyrosine kinase of the TAM family (TYRO3, AXL, MERTK), which has emerged as a critical regulator of tumor progression and immune evasion. AXL can be activated by its ligand GAS6 or through ligand-independent mechanisms, leading to the promotion of oncogenic processes, including proliferation, invasion, metastasis, epithelial–mesenchymal transition (EMT), angiogenesis, stemness, and therapy resistance [10]. AXL is overexpressed in several cancers, like lung, liver, renal, pancreatic, and ovarian cancers, especially in TNBC tumors, where it is associated with poor clinical outcomes [11,12]. Notably, AXL overexpression in TNBC has been associated with chemotherapy resistance and a higher risk of relapse, indicating its potential as a therapeutic target [13]. Beyond its role in tumor-intrinsic biology, AXL also influences the tumor microenvironment. AXL signaling has been shown to suppress natural killer (NK) cell activity, diminish antitumor immune responses, and promote a protumorigenic niche [14]. This dual role, supporting both tumor cell survival and immune evasion, makes AXL an appealing target for therapeutic intervention.

In light of AXL’s central role in TNBC progression, immune evasion, and chemoresistance, pharmacologic AXL inhibition represents a promising therapeutic strategy. AB-329 (formerly DS-1205b), a selective small-molecule AXL inhibitor, has demonstrated safety and preliminary efficacy in combination with EGFR inhibitors in clinical trials for non-small cell lung cancer (NSCLC) [15,16]. However, its role in TNBC remains unexplored. Given the urgent need for targeted strategies that can overcome chemoresistance and stimulate immune responses, this study investigates AB-329 as a novel approach to treating AXL-overexpressing TNBC. We evaluated AB-329 in preclinical TNBC models to assess its impact on tumor growth, metastasis, and immune modulation both as monotherapy and in combination with chemotherapy. Our findings provide preclinical justification for clinical translation of AXL-targeted therapy in TNBC.

## 2. Results

### 2.1. AXL Expression Is Elevated in TNBC and Targetable by an AXL-Specific Inhibitor, AB-329

**Figure 1:**
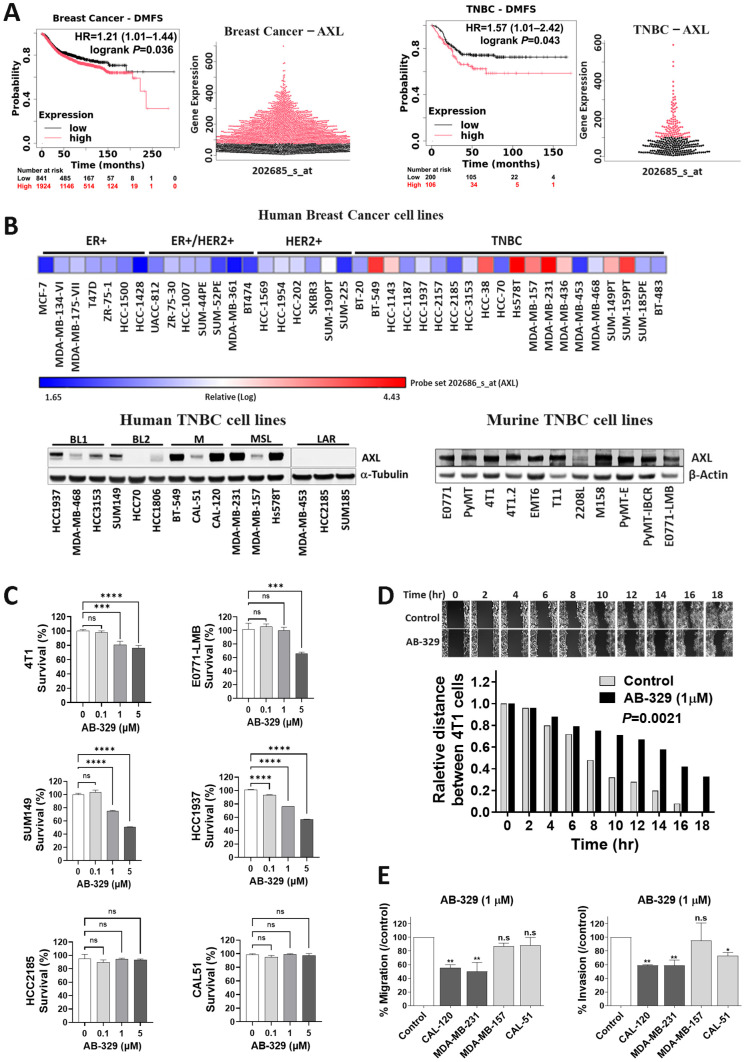
AXL is overexpressed in TNBC and predicts poor clinical outcomes. (**A**) Distant-metastasis-free survival (DMFS) was evaluated through Kaplan–Meier analysis of 2765 breast cancer and 306 TNBC patients using the KM Plotter database. (**B**) AXL mRNA expression across 40 breast cancer cell lines was analyzed using the ArrayExpress dataset E-TABM-157 https://www.ebi.ac.uk/arrayexpress/experiments/E-TABM-157 (accessed 15 March 2017). AXL protein levels were assessed through Western blot in human TNBC subtype cell lines BL1 (basal-like 1), BL2 (basal-like 2), M (mesenchymal), MSL (mesenchymal stem-like), and LAR (luminal AR) and murine TNBC cell lines. (**C**) AXL is targetable by an AXL-specific inhibitor, AB-329. SRB proliferation assay was conducted using human and murine TNBC cell lines. Clonogenic assay. (**D**) Wound healing assay showing the time-dependent effect of AB-329 on the migration of murine TNBC cells. (**E**) Evaluation of the inhibitory effect of AB-329 on TNBC cell motility using Transwell migration and invasion assays. Data are presented as mean ± SEM (*n* = 3 per group). Proliferation, migration, and invasion data were analyzed using one-way ANOVA followed by Tukey’s test, while the wound healing assay used an unpaired *t*-test. Statistical significance is indicated as ns/n.s (not significant), * *p* ≤ 0.05, ** *p* ≤ 0.01, *** *p* ≤ 0.001, and **** *p* ≤ 0.0001.

Dysregulated AXL expression has been linked to increased tumor progression and metastasis and impaired tumor immunogenicity. To assess the prognostic significance of AXL, we analyzed 3911 breast cancer and 267 TNBC patients using the Kaplan–Meier plotter. The result showed that high AXL expression is associated with poorer distant-metastasis-free survival (DMFS) in both breast cancer (*p* = 0.036) and TNBC (*p* = 0.043, Figure 1A). Next, we assessed AXL mRNA and protein expression levels in a set of breast cancer cell lines. As shown in Figure 1B, AXL mRNA expression levels were relatively low in hormone-positive and HER2-positive breast cancer cells lines. In contrast, most TNBC cell lines exhibited significantly elevated AXL mRNA expression levels. Similarly, AXL protein expression was notably higher in human TNBC cells and mouse TNBC cell line models (Figure 1B), particularly in mesenchymal subtypes compared to other breast cancer cell types.

The antiproliferative activity of AB-329 was assessed in vitro across TNBC cell lines with varying AXL expression using a sulforhodamine B (SRB) assay. AB-329 exhibited moderate growth-inhibitory effects in AXL-positive human (SUM149, HCC1937) and murine (4T1, E0771-LMB) TNBC cells, with half-maximal inhibitory concentrations (IC50) exceeding 5 μM (Figure 1C). In contrast, no significant activity was observed in AXL-negative TNBC cell lines (HCC2185, CAL51), underscoring the dependence of AB-329 efficacy on AXL expression. Among the AXL-positive lines, 4T1, E0771-LMB, and SUM149 showed the highest AXL levels and greatest sensitivity and were therefore selected for subsequent studies. Given the association between elevated AXL expression and reduced distant-metastasis-free survival (DMFS) in breast cancer and TNBC patients (Figure 1A), we next investigated the impact of AB-329 on cell motility. AB-329 significantly suppressed the migration of murine TNBC cells (4T1, *p* = 0.0021) in a time-dependent manner in wound-healing assays (Figure 1D) and inhibited both migration (MDA-MB-231; *p* = 0.0053, CAL120; *p* = 0.0085) and invasion (MDA-MB-231; *p* = 0.0037, CAL120; *p* = 0.0073) of AXL-high human TNBC cells while exerting minimal effects on AXL-low lines (MDA-MB-157, CAL51) (Figure 1E). Collectively, these results highlight AXL as a promising therapeutic target in metastatic TNBC and demonstrate the selective activity of AB-329 against AXL-positive tumors.

### 2.2. AB-329 Combined with Paclitaxel Synergistically Inhibited the Proliferation, Migration, and Invasion of TNBC Cells

**Figure 2:**
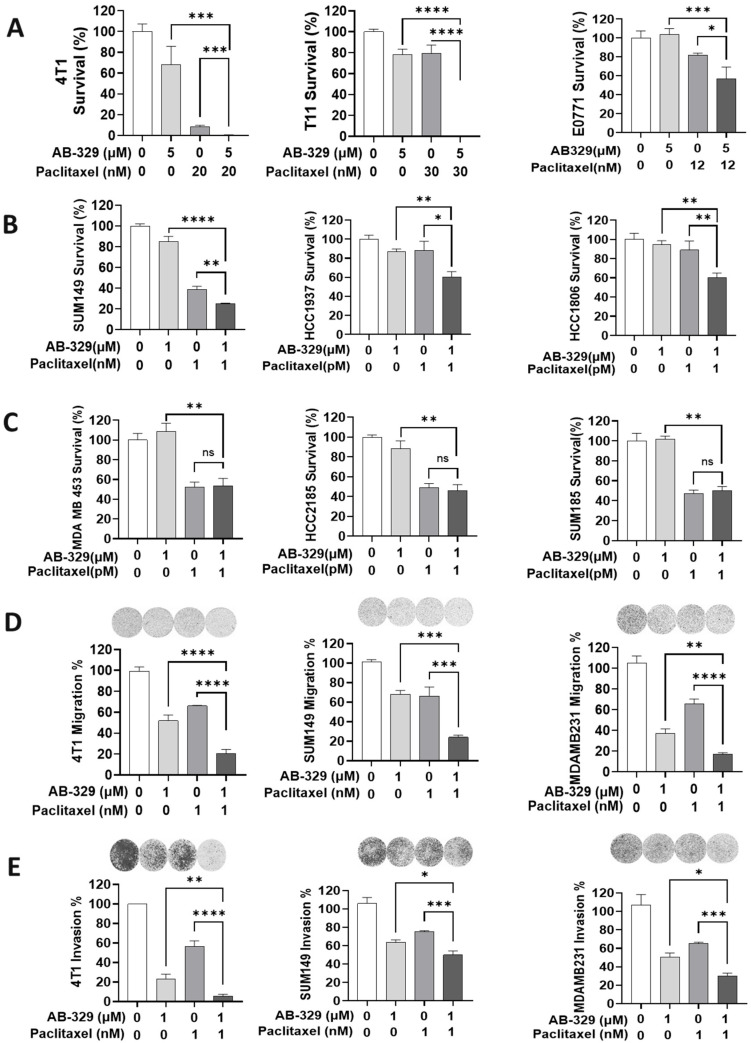
AB-329 synergizes with paclitaxel to inhibit TNBC cell proliferation, migration, and invasion. (**A**–**C**) Clonogenic assay in AXL-positive and AXL-negative human TNBC cells and murine TNBC cell lines. (**D**) Migration assays were conducted in a 24-well microchemotaxis chamber with 8 µm pore size inserts and human, and murine TNBC cells were permitted to migrate for 6 to 8 h. (**E**) Invasion assays were conducted using a 24-well Transwell chamber, following the same procedure as the migration assays, except that the inserts were pre-coated with 100 µL of Matrigel (diluted 1:15 in FBS-free medium), and cells were allowed to invade for 24 h. Data are presented as mean ± SEM (*n* = 3 per group) and were analyzed using a two-tailed unpaired Student’s *t*-test. Statistical significance is indicated as ns (not significant), * *p* ≤ 0.05, ** *p* ≤ 0.01, *** *p* ≤ 0.001, and **** *p* ≤ 0.0001.

Given the moderate antiproliferative activity of AB-329 and its dependency on AXL expression, we next explored whether combining AB-329 with chemotherapy could enhance its antitumor effects in AXL-positive TNBC models. We screened various chemotherapy agents like paclitaxel (Figure 2 and Figure S1), Gemcitabine, DXd, and SN-38 (Figure S2). The paclitaxel combination therapy significantly suppressed colony formation by more than 50% in murine TNBC cells (4T1 cells (*p* = 0.0008), T11 cells (*p* = 0.0001), and EO771 cells (*p* < 0.0215, Figure 2A) and by 20 to 40% in human TNBC cells (SUM149 cells (*p* < 0.012), HCC1937 cells (*p* < 0.012), and HCC1806 cells (*p* < 0.0094, Figure 2B). However, the combination of AB-329 and paclitaxel did not exhibit synergistic activity in AXL-negative TNBC cell lines (MDAMB453, HCC2185 and SUM185, Figure 2C), suggesting that AB-329′s synergistic activity relies on AXL expression levels. Synergy between AB-329 and chemotherapeutic agents was evaluated using the Bliss independence model. A positive ΔBliss score shows synergy (4T1 cells = 5.02, T11 cells = 61.7, EO771 cells = 27.9, SUM149 cells = 7.8, HCC1937 cells = 16.1, HCC1806 cells = 23.4), while a negative ΔBliss indicates antagonism (MDAMB453 = −1.5, HCC2185 = −2.7, and SUM185 = −1.9). The AB-329 and paclitaxel combination significantly decreased TNBC cell proliferation compared to monotherapy, prompting us to investigate whether this combination affects TNBC cell migration and invasion. The paclitaxel and AB-329 combination reduced migration by over 50% in both murine and human TNBC cell lines (4T1, *p* = 0.0001; SUM149, *p* = 0.0002; and MDA-MB-231, *p* = 0.0001, Figure 2D), and invasion decreased by more than 50% with paclitaxel in 4T1 cells (*p* = 0.0001) and approximately 15 to 20% in SUM149 (*p* = 0.0004) and MDA-MB-231 cells (*p* = 0.0006) (Figure 2E). Combination therapy significantly inhibited TNBC cell proliferation, migration, and invasion compared to monotherapy, suggesting that targeting AXL enhances chemotherapeutic efficacy in AXL-positive TNBC.

### 2.3. AB-329 Combined with Paclitaxel Synergistically Reduced Tumor Growth in Murine TNBC Models

**Figure 3:**
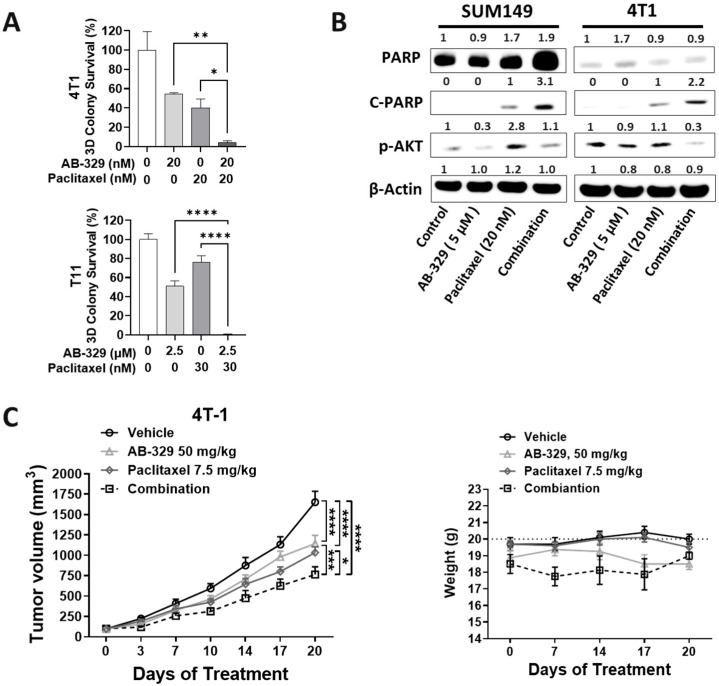

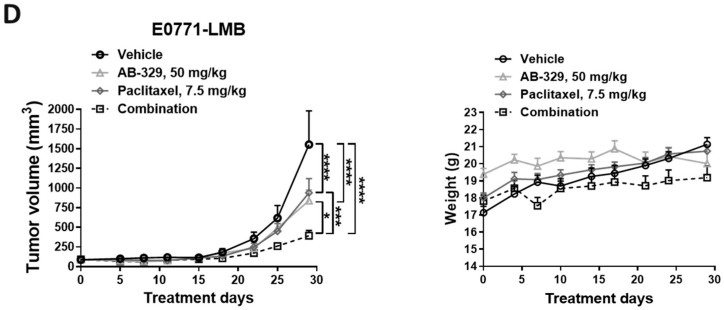
AB-329 synergizes with paclitaxel in murine TNBC xenograft models. (**A**) Soft agar assay was performed using 4T1 cells and T11 cells treated with AB-329 in combination with paclitaxel. (**B**) SUM149 and 4T1 cells were treated with AB-329 and paclitaxel for 48 h, total cell protein was collected, and 20 µg of protein was used to run Western blots. (**C**,**D**) Xenograft assay using 4T1 and E0771-LMB. Tumors were induced by injecting cells into the mammary fat pad of mice, and treatments were initiated once tumors reached an average of 100–150 mm^3^. AB-329 (50 mg/kg) was administered once a day via oral gavage. Paclitaxel was administered at a dose of 7.5 mg/kg via tail vein injection once per week. Body weights of 4T1 and E0771-LMB mouse models. In vivo data are presented as mean ± SEM (*n* = 6–10 per group) and were analyzed using multiple *t*-tests with correction for multiple comparisons. Statistical significance is indicated as ns (not significant), * *p* ≤ 0.05, ** *p* ≤ 0.01, *** *p* ≤ 0.001, and **** *p* ≤ 0.0001.

Building on the observed in vitro synergy between AB-329 and paclitaxel, we next assessed their combined antitumor effectiveness in vivo using murine TNBC xenograft models. In vitro, AXL inhibition boosted the growth-suppressive effect of paclitaxel, which was further confirmed in 3D soft agar assays (4T1 cells (*p* = 0.014), T11 cells (*p* = 0.0001)) (Figure 3A). Mechanistically, the combination increased apoptotic c-PARP expression and decreased p-AKT signaling (Figure 3B and Figure S3A), indicating enhanced induction of apoptosis. To evaluate this effect in vivo, mice bearing 4T1 or E0771-LMB tumors were treated with either vehicle, AB-329 or paclitaxel, or a combination of both. AB-329 (*p* < 0.0001) and paclitaxel (*p* < 0.0001) significantly suppressed tumor growth compared to the control group. Notably, the combination achieved superior tumor suppression compared with either monotherapy (*p* < 0.001 vs. AB-329; *p* < 0.05 vs. paclitaxel) (Figure 3C,D). Importantly, the combination was well-tolerated, with no significant changes in body weight observed. Overall, these results demonstrate that AB-329 works synergistically with paclitaxel to improve antitumor efficacy in AXL-positive TNBC models.

### 2.4. AXL Inhibition Enhances Activated NK Cell Infiltration in Breast Cancer Tissues

**Figure 4:**
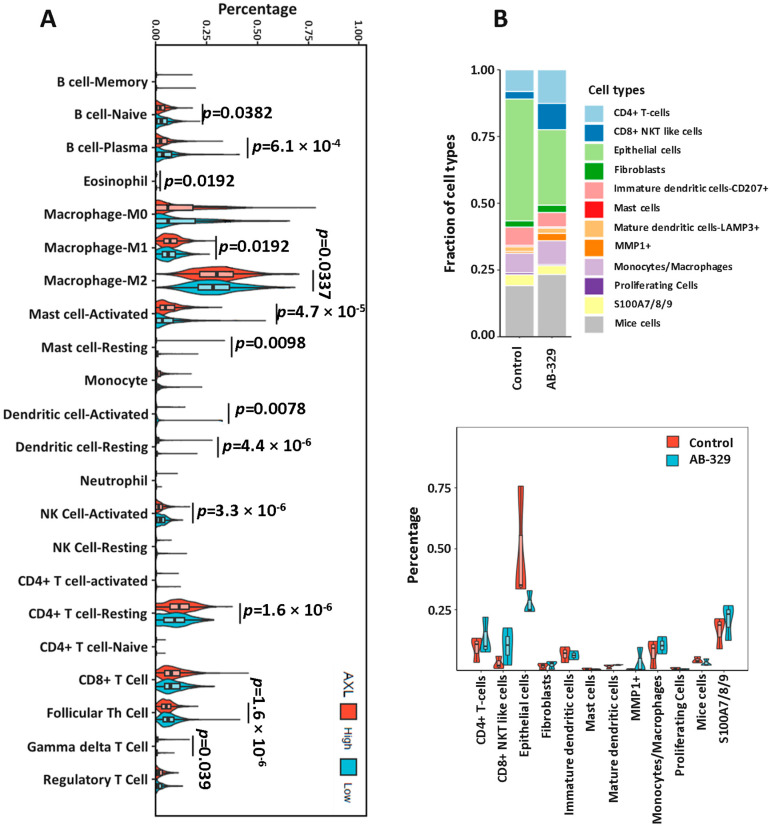
AXL inhibition improved activated NK cell numbers in breast cancer tissues. (**A**) The immune cell fraction in 1200 patients with breast cancer was analyzed using The Cancer Genome Atlas (TCGA) data. (**B**) AXL effect on human NK cells was analyzed using the SUM149 humanized mouse model treated with only AB-329 through single-cell RNA sequencing.

Beyond its established role in driving tumor proliferation, migration, and metastasis, AXL also promotes immune evasion by modulating the tumor microenvironment. Thus, we next sought to determine whether AXL inhibition could modulate the tumor immune microenvironment (TME). Specifically focusing on innate immune responses, we analyzed the immune cell fraction in 1200 patients with breast cancer in The Cancer Genome Atlas (TCGA). The data showed that low AXL expression was associated with highly activated NK cells (*p* < 0.0001, Figure 4A). To confirm the AXL effect on human NK cells, we used the SUM149 xenograft humanized mice model treated with only AB-329, which showed a similar kind of result in the immune cell fraction based on single-cell RNA sequencing (Figure 4B). Our results collectively indicate that targeting AXL using AB-329 improves the tumor immuno-microenvironment by increasing activated NK cells.

## 3. Discussion

Triple-negative breast cancer (TNBC) remains a major clinical challenge due to its aggressive behavior, molecular heterogeneity, and limited treatment options. In this study, we present preclinical evidence supporting the therapeutic potential of AB-329, a selective AXL kinase inhibitor, in AXL-expressing TNBC. Our data show that among all breast cancer subtypes, TNBC exhibits the highest levels of AXL expression and a strong association between AXL overexpression and the mesenchymal subtype of TNBC, which is known for its increased metastatic potential and resistance to chemotherapy [17,18,19]. We demonstrate that AB-329 significantly inhibits tumor growth and metastasis while reprogramming the tumor immune microenvironment to promote antitumor immunity, primarily through enhanced infiltration of activated NK cells. These findings highlight AXL’s dual role in mediating both tumor-intrinsic aggressiveness and tumor-extrinsic immune suppression and support AXL inhibition as a strategy to overcome two major mechanisms of therapeutic resistance in TNBC.

AB-329 monotherapy inhibited the proliferation and migration of TNBC cells, consistent with AXL’s known role in promoting epithelial–mesenchymal transition and stem-like phenotypes. Western blot analysis confirmed the effective inhibition of downstream AKT signaling (Figure S3A), validating the on-target activity of AB-329. Additionally, Gene Set Enrichment Analysis (GSEA) of tumor cells revealed that AB-329 induced the activation of immune and stress-related pathways (e.g., JAK/STAT3, apoptosis, TNF/NF-κB) while suppressing hypoxia and immunosuppressive signaling (Figure 3B). GSEA of NKT cells further showed increased expression of interferon and apoptosis pathways, accompanied by downregulation of cholesterol homeostasis and IL6/JAK/STAT3 signaling (Figure S3C), suggesting that AB-329 reprograms immune metabolism to enhance antitumor function.

To optimize combination strategies, we tested several chemotherapeutic agents, including paclitaxel, gemcitabine, DXd, and SN-38, with AB-329 using both 2D (Figure 2A–C and Figure S2A) and 3D clonogenic assays (Figure 3A and Figure S2B). Among these, paclitaxel and SN-38 showed the most consistent and synergistic growth inhibition when combined with AB-329, especially in the 3D format, which better mimics in vivo tumor architecture. Notably, SN-38 is the active metabolite of irinotecan. However, our previous in vivo studies indicated that AB-329 did not synergize with irinotecan. These findings led us to focus on the paclitaxel + AB-329 combination for further investigation.

At the molecular level, our data suggest that AB-329, when combined with paclitaxel, disrupts pro-survival signaling by downregulating phosphorylated AKT (p-AKT), a critical mediator of cell survival and proliferation. This was accompanied by elevated levels of cleaved PARP, indicating enhanced apoptosis and chemosensitivity. Functionally, the combination produced synergistic antitumor effects in murine models, with greater tumor regression and increased infiltration of activated NK cells compared to either agent alone. Supporting this mechanism, TCGA analysis revealed that low AXL expression correlates with higher levels of activated NK cells, highlighting an inverse relationship between AXL signaling and antitumor immunity. This synergy is mechanistically plausible, as paclitaxel-induced immunogenic cell death increases tumor visibility to the immune system, while AXL inhibition relieves immune suppression and promotes NK cell activation.

Given that metastasis accounts for approximately 90% of cancer-related deaths [20], targeting AXL-driven migration and invasion is a critical therapeutic objective. Our data demonstrate that AB-329 significantly suppresses TNBC cell migration and invasion, with further enhancement when combined with paclitaxel, underscoring its potential to mitigate metastasis and overcome chemoresistance. These findings provide a strong rationale for combining AXL inhibitors with paclitaxel chemotherapy in TNBC. Importantly, analysis of clinical datasets revealed that high AXL expression is significantly associated with worse distant-metastasis-free survival (DMFS) in both breast cancer and TNBC, further supporting the clinical relevance of AXL as both a prognostic biomarker and a therapeutic target.

In the context of AXL-targeted therapies, several inhibitors have advanced into clinical trials, including Bemcentinib (BGB324) [21], Dubermatinib (TP-0903) [22], and Sitravatinib (MGCD516) [23]. While these agents have shown varying degrees of efficacy across cancers, their limitations include broader kinase inhibition profiles, suboptimal pharmacodynamics, or toxicity-related concerns that can complicate combination regimens. For example, Sitravatinib and Dubermatinib target multiple kinases beyond AXL (including MET, VEGFR, and FLT3), which can result in off-target toxicities. Bemcentinib, although more selective, has demonstrated variable potency in AXL-high solid tumors, and its performance in combination with chemotherapy in TNBC remains unclear.

While these findings support the further development of AB-329, several questions remain. The precise mechanisms through which AXL inhibition enhances NK cell activity are not fully understood. Future studies should investigate changes in cytokines and chemokines, AXL’s role in myeloid cells, and combination strategies with immune checkpoint inhibitors. Additionally, preclinical studies are necessary to confirm whether AXL expression or immune contexture can act as predictive biomarkers of AB-329’s response. In summary, our study provides strong preclinical evidence that AB-329 is a promising candidate for combination therapy in AXL-positive TNBC. By targeting both intrinsic tumor cell pathways and the immunosuppressive tumor microenvironment, AB-329 may overcome chemotherapy resistance and enhance antitumor immunity. These findings support the rationale for evaluating AB-329 in clinical trials for advanced TNBC.

## 4. Materials and Methods

### 4.1. Cell Lines and Reagents

Murine TNBC cell lines included 4T1 (purchased from the American Type Culture Collection [ATCC], Manassas, VA, USA), E0771 and E0771-LMB (obtained from a collaborator), and T11 (Baylor College of Medicine). Human TNBC cell lines included SUM149 and SUM185 (BIOIVT, Westbury, NY, USA), HCC1937, HCC1806, MDA-MB-231, MDA-MB-157, and MDA-MB-453 (ATCC), as well as HCC2185, CAL51, and CAL120 (obtained from collaborators). 4T1, T11, MDA-MB-231, MDA-MB-157, and MDA-MB-453 cells were cultured in Dulbecco’s Modified Eagle Medium/F12 (DMEM/F12; #D8062; Sigma-Aldrich, St. Louis, MO, USA). E0771-LMB, HCC1937, HCC1806, HCC2185, CAL51, and CAL120 cells were maintained in RPMI-1640 medium (#R8758; Sigma-Aldrich, St. Louis, MO, USA). Both DMEM/F12 and RPMI media were supplemented with 10% fetal bovine serum (FBS; #F0600-050; Gen-DEPOT, Katy, TX, USA) and 1% antibiotic/antimycotic (#A5955; Sigma-Aldrich, St. Louis, MO, USA). SUM149 and SUM185 cells were cultured in Ham’s F-12 medium (#11765054; Invitrogen, Waltham, MA, USA) supplemented with 10% FBS, 1% antibiotic/antimycotic, insulin (5 µg/mL; #12-585-014; Thermo Fisher Scientific, Waltham, MA, USA), and hydrocortisone (1 µg/mL; #H0888; Sigma-Aldrich, St. Louis, MO, USA). All cell lines were authenticated by the Characterized Cell Line Core Facility at The University of Texas MD Anderson Cancer Center using short tandem repeat (STR) profiling and confirmed to be free of mycoplasma contamination using the MycoAlert Mycoplasma Detection Kit (#LT07-710; Lonza, Morristown, NJ, USA). AB-329 was kindly provided by AnHeart Therapeutics (New York, NY, USA).

### 4.2. Clonogenic Assay

Clonogenic assay was used to evaluate the effects of drug treatments on cancer cell growth. First, 2–5 × 10^4^ cells were seeded per well in 6-well plates and cultured overnight. The following day, cells were treated with AB-329, paclitaxel, or a combination of AB-329 and paclitaxel and incubated at 37 °C for up to 10 days. After treatment, cells were fixed with 5% trichloroacetic acid (Sigma-Aldrich, St. Louis, MO, USA) for two hours and stained with 0.03% sulforhodamine B (Sigma-Aldrich, St. Louis, MO, USA) for 30 min at room temperature. Excess stain was removed by washing three times with 1% acetic acid, and the bound dye was solubilized in 10 mM Tris buffer (Bio-Rad, Hercules, CA, USA). Fluorescence was measured at 480 nm (Ex)/590 nm (Em) using a VICTOR X3 plate reader (PerkinElmer, Waltham, MA, USA) or a Spark microplate reader (Tecan, Männedorf, Switzerland).

### 4.3. Soft Agar Assay

The effect of drug treatment on the anchorage-independent growth of 4T1 and T11 cells was assessed using a soft agar assay. Cells (5 × 10^3^ cells/well) were suspended in 0.375% agarose (#16520050; Thermo Fisher Scientific., Waltham, MA, USA) in a complete medium containing drugs. Colonies were grown on 6-well plates with a 0.75% agarose layer and incubated for up to 2 weeks. Following treatment, the colonies were stained with MTT (3-(4,5-dimethylthiazol-2-yl)-2,5-diphenyltetrazolium bromide; #M5655; Sigma-Aldrich, St. Louis, MO, USA) for two hours. The number of colonies exceeding 80 μm in diameter was then determined using a GelCount system (Oxford Optronix, Adderbury, England).

### 4.4. Gene Expression

AXL gene (Probeset: 202686_s_at) expression analysis was performed using a BioGPS dataset of forty breast cancer cell lines.

### 4.5. Western Blot

First, 1–2 × 10^6^ cells per 10 mL were seeded into 10 cm plates and cultured overnight. Whole cell lysate was isolated using M-PER Mammalian Protein Extraction Reagent (#78501; Thermo Fisher Scientific) supplemented with phosphatase and protease inhibitors (#P3300-001; Gendepot). Protein samples were then analyzed through Western blotting. Membranes were probed with the following primary antibodies at 1:1000 dilution: anti-human AXL (#5153), Phospho-Akt (Ser473) (#9271), Akt (#4691), PARP (#9542) (all from Cell Signaling Technology, Danvers, MA, USA), anti-mouse AXL (#AF854; R&D Systems), and β-actin (#A5316; Sigma-Aldrich, St. Louis, MO, USA). Horseradish peroxidase-conjugated secondary antibodies (1:10,000; Invitrogen) were used for chemiluminescent detection.

### 4.6. Cell Migration Assay and Cell Invasion Assay

Cell migration was assessed using 24-well Transwell chambers (8 µm pores; Corning Inc., Corning, NY, USA). Cells (2.5 × 10^5^ in 250 µL FBS-free medium) were seeded into the upper chambers, while the lower chambers were filled with 750 µL of complete medium containing 10% FBS as a chemoattractant. After 6–8 h, migrated cells were fixed and stained with hematoxylin and eosin. For invasion assays, the upper chambers were pre-coated with 100 µL of Matrigel diluted 1:15 in FBS-free medium, and cells were allowed to invade for 24 h. Images were captured at 10× magnification using an Eclipse 80i microscope (Nikon Instruments Inc., Melville, NY, USA), and migrated or invaded cells were quantified by counting four randomly selected fields per insert.

### 4.7. TNBC Mouse Model

All animals were housed and handled following the guidelines of the MD Anderson Institutional Animal Care and Use Committee. 4T1 (2 × 10^4^/100 µL) and E0771-LMB (2 × 10^5^/100 µL) cells were implanted into one of the mammary fat pads of 4- to 6-week-old female BALB/c. When tumor size reached 50–100 mm3, mice were randomly divided into four groups (10 mice/group) and treated with vehicle or AB-329 at 50 mg/kg/day orally daily or paclitaxel at 7.5 mg/kg/week via intravenous injections weekly once or both AB-329 and paclitaxel treatments for 30 days. At the end of the study, tumor samples were collected, fixed, and processed for immunohistochemical (IHC) staining to evaluate the expression of target proteins.

### 4.8. Immune Cell Fraction in SUM149 Humanized Mice Model

Myeloablated NSG-SGM3 mice were engrafted with CD34+ human hematopoietic stem cells to generate CD34+ humanized mice. Successful human immune cell engraftment was confirmed by detecting >25% human CD45+ (hCD45+) cells in peripheral blood via fluorescence-activated cell sorting (FACS). SUM149 cells (4 × 10^6^) were implanted into a mammary fat pad of each hCD45+ humanized mouse. When tumors reached an average volume of 100 mm^3^, mice were randomly assigned to two groups (10 mice per group) and treated with either vehicle or AB-329 (50 mg/kg) administered orally once daily. At the study’s endpoint, tumors were collected, processed into single-cell suspensions, and prepared for single-cell RNA sequencing analysis.

### 4.9. Human Breast Cancer TCGA Data

Gene expression data for TCGA breast cancer (BRCA) samples were obtained from the UCSC Xena platform, and immune infiltration estimates for these tumors were retrieved from the TIMER2.0 database. BRCA samples were stratified into two groups based on the median expression of the AXL gene. Differences in immune cell proportions between the high-AXL and low-AXL groups were assessed across all immune cell types from CIBERSORT using the Wilcoxon rank-sum test and Student’s *t*-test.

### 4.10. Statistical Analysis

Cell proliferation, migration, and invasion rates were summarized as mean ± standard deviation. Statistical comparisons for in vitro experiments were performed using a two-tailed unpaired Student’s *t*-test in GraphPad Prism 9. For xenograft studies, treatment groups were compared at specified time points using multiple *t*-tests with correction for multiple comparisons. A *p*-value ≤ 0.05 was considered statistically significant.

## Data Availability

The corresponding authors will provide the raw data supporting the conclusions of this article upon request.

## Supplementary Materials

The following supporting information can be downloaded at https://www.mdpi.com/article/10.3390/ijms26188896/s1.

## Abbreviations

Here are the abbreviations used in this manuscript:

TNBC	Triple-negative breast cancer
EMT	Epithelial–mesenchymal transition
NK	Natural killer
ER	Estrogen receptor
PR	Progesterone receptor
HER-2	Human epidermal growth factor receptor 2
BL1	Basal-like 1
BL2	Basal-like 2
M	Mesenchymal
MSL	Mesenchymal stem-like
IM	Immunomodulatory
LAR	Luminal androgen receptor
TAM	TYRO3-AXL-MERTK
GAS6	Growth arrest-specific protein 6
DMFS	Distant-metastasis-free survival
TCGA	The Cancer Genome Atlas
CSC	Cancer stem cell
