# Supplementary material for: Dual Therapeutic Impact of AXL Inhibitor AB-329: Chemotherapy Sensitization and Immune Microenvironment Reprogramming in TNBC
Source: Int J Mol Sci. 2025 Sep 12;26(18):8896. doi: 10.3390/ijms26188896 (PMC12469809; doi:10.3390/ijms26188896)
Supplement: Supplementary file 1 [file ijms-26-08896-s001.zip › ijms-3778222-supplementary.pdf]

A

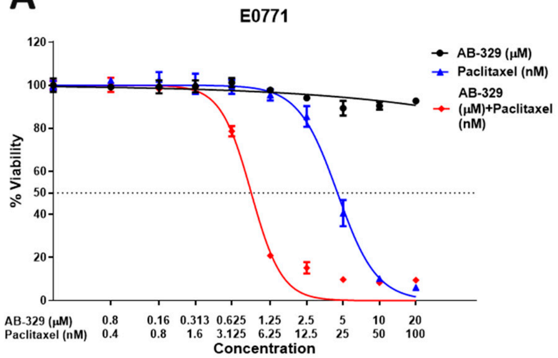

CI For experimental values

AB-329 (µM)	PTX (nM)	Fa	CI
0.625	3	0.213759	0.155
1.25	6.25	0.790941	0.169
2.5	12.5	0.847842	0.308
5	25	0.902052	0.548
10	50	0.916003	1.055
20	100	0.905018	2.176

B

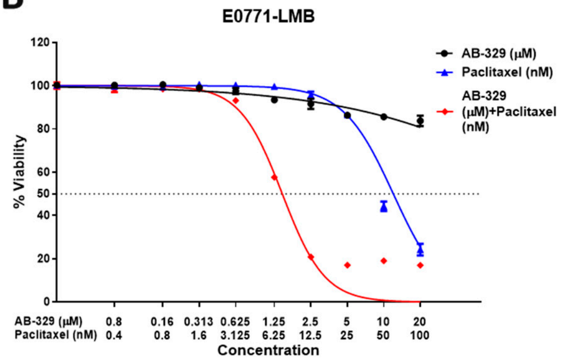

CI For experimental values

AB-329 (µM)	PTX (nM)	Fa	CI
1.25	6.25	0.42308	0.190
2.5	12.5	0.791523	0.189
5	25	0.828839	0.342
10	50	0.809213	0.722
20	100	0.82964	1.363

C

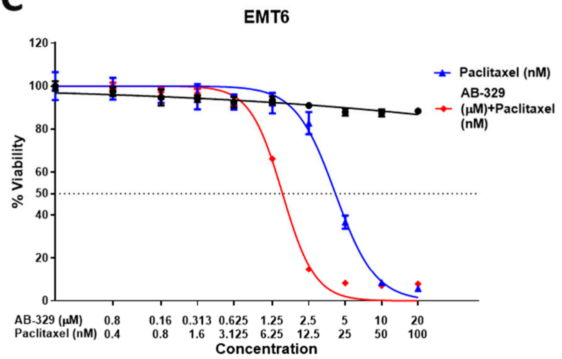

CI For experimental values

AB-329 (µM)	PTX (nM)	Fa	CI
1.25	6.25	0.33854	0.657
2.5	12.5	0.852668	0.182
5	25	0.917285	0.214
10	50	0.929672	0.371
20	100	0.921475	0.818

D

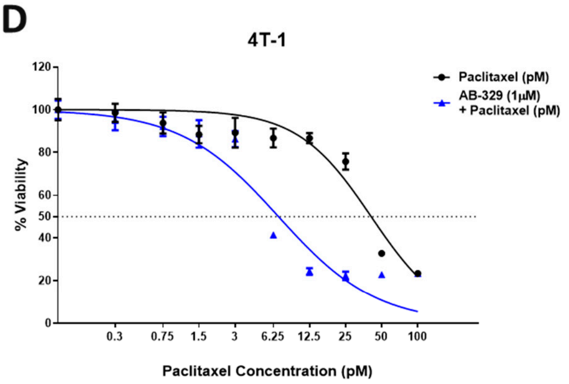

CI For experimental values

Paclitaxel (nM)	AB-329 (µM)	Fa	CI
6.25	1.00	0.32	0.04
12.50	1.00	0.63	0.03
25.00	1.00	0.67	0.05
50.00	1.00	0.59	0.13

Supplementary Figure 1. AB-329 synergizes with Paclitaxel. AB-329 increases the growth-inhibiting effects of Paclitaxel in murine TNBC cell lines **(A)** E0771, **(B)** E0771-LMB, **(C)** EMT6, and **(D)** 4T-1, as measured by a sulforhodamine B staining assay after a 5-day treatment. Data are presented as mean ± SEM (n = 3 per group) and Combinational index (CI) value: <0.1 indicates very strong synergism; 0.10-0.30, strong synergism; 0.31-0.70, synergism; 0.71-0.85, moderate synergism; 0.86-0.90, slight synergism; 0.91-1.10, nearly additive; 1.11-1.20, slight antagonism; 1.21-1.45, moderate antagonism; 1.46-3.30, antagonism; 3.31-10, strong antagonism; >10, very strong antagonism. Fa, fractional index.

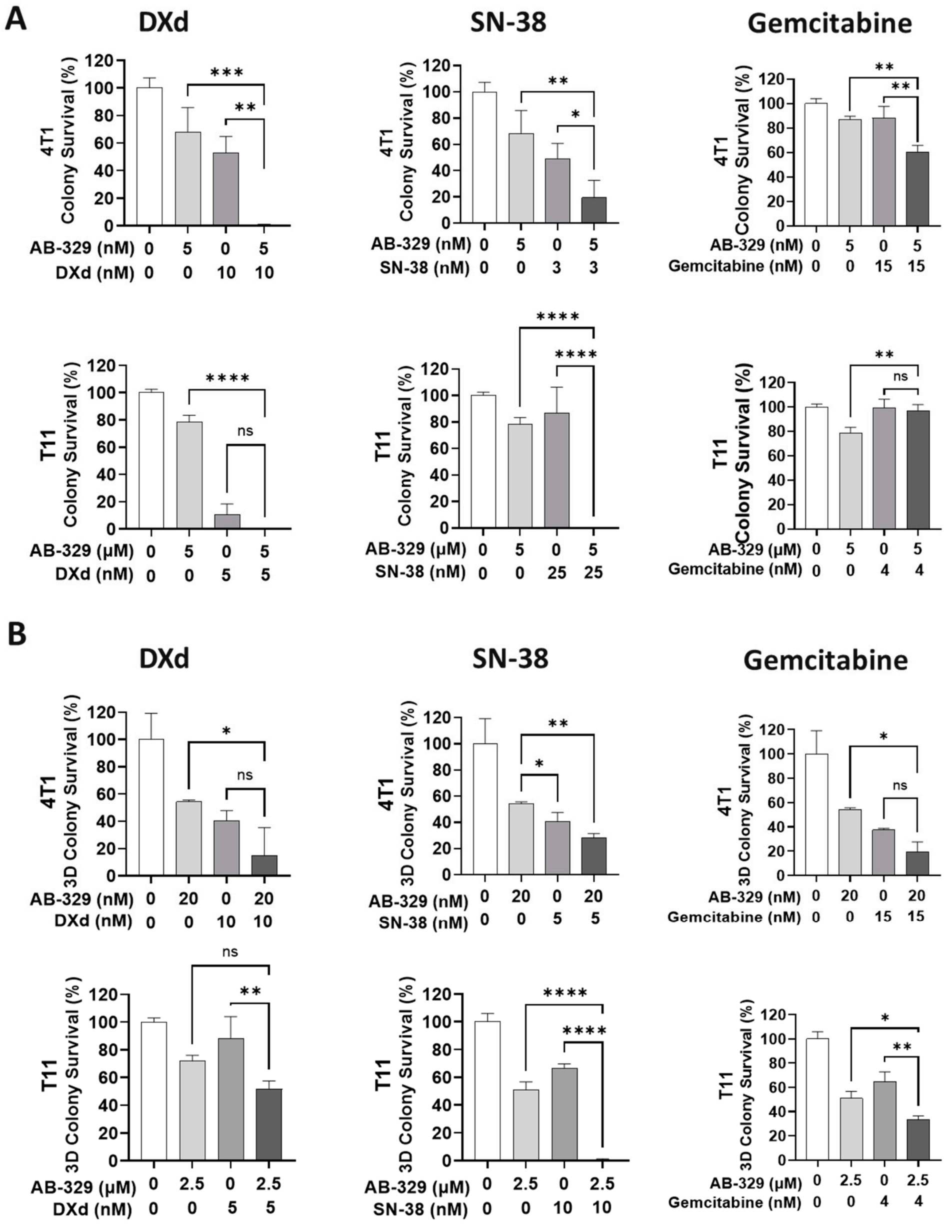

Supplementary Figure 2. AXL inhibition improved chemotherapy efficacy in murine TNBC cells. **(A)** Clonogenic assay was performed using 4T1 cells and T11 cells treated with AB-329 in combination of DXd, SN38, and Gemcitabine. **(B)** Soft agar assay was performed using 4T1 cells and T11 cells treated with AB-329 in combination of DXd, SN38, and Gemcitabine. Data are presented as mean ± SEM (n = 3 per group) and were analyzed using a two-tailed unpaired Student's t-test, and statistical significance is indicated as ns (not significant), * P ≤ 0.05, ** P ≤ 0.01, *** P ≤ 0.001, and **** P ≤ 0.0001.

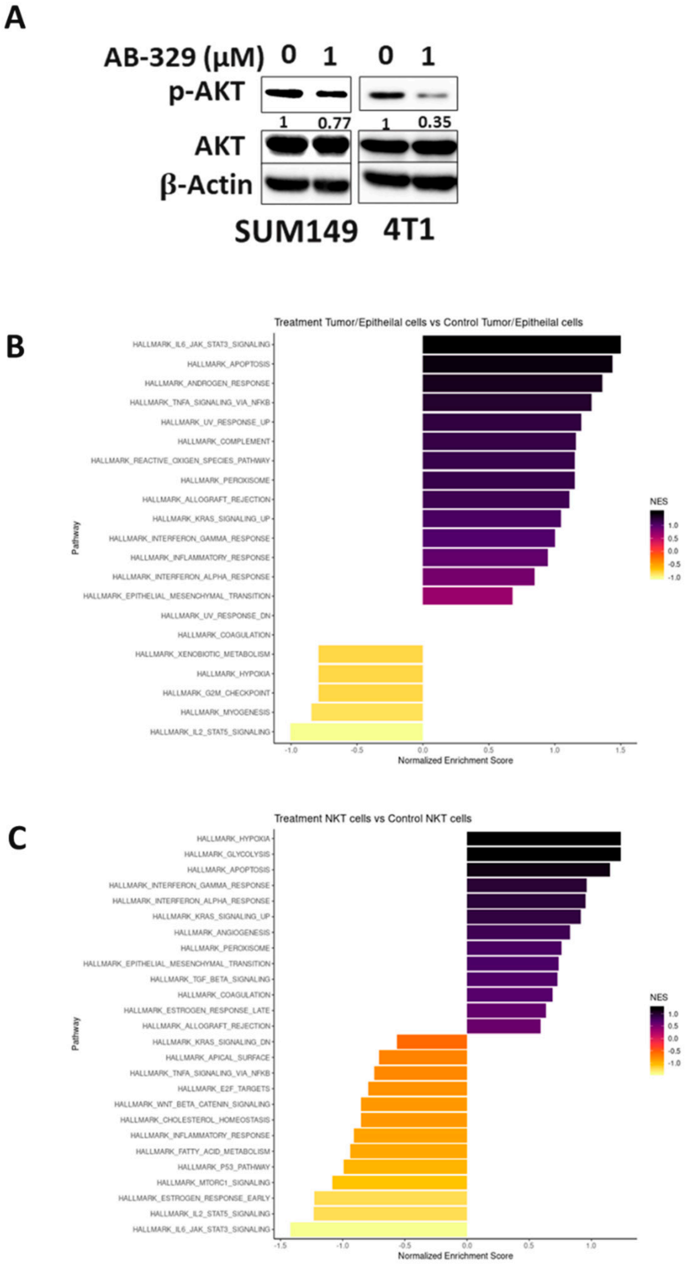

Supplementary Figure 3. AXL inhibition effect on cell signaling pathways in TNBC **(A)** shows that AB329 treatment reduced p-AKT levels in both murine and human TNBC cell lines. The impact of AB-329 on cell signaling pathways in cancer epithelial cells **(B)** and NK cells **(C)** from the SUM149 humanized mouse model was analyzed using single-cell RNA sequencing.
